# Evidence of *Bos javanicus* x *Bos indicus* hybridization and major QTLs for birth weight in Indonesian Peranakan Ongole cattle

**DOI:** 10.1186/s12863-015-0229-5

**Published:** 2015-07-04

**Authors:** Hartati Hartati, Yuri Tani Utsunomiya, Tad Stewart Sonstegard, José Fernando Garcia, Jakaria Jakaria, Muladno Muladno

**Affiliations:** Beef Cattle Research Station, Indonesian Agency for Agricultural Research and Development, Ministry of Agriculture, Jln. Pahlawan no. 2 Grati, Pasuruan, East Java 16784 Indonesia; Faculdade de Ciências Agrárias e Veterinárias, UNESP - Univ Estadual Paulista, Jaboticabal, São Paulo 14884-900 Brazil; ARS-USDA - Agricultural Research Service - United States Department of Agriculture, Animal Genomics and Improvement Laboratory, Beltsville, MD 20705 USA; Faculdade de Medicina Veterinária de Araçatuba, UNESP – Univ Estadual Paulista, Araçatuba, São Paulo 16050-680 Brazil; Faculty of Animal Science, Bogor Agriculture University, Jln. Agatis kampus IPB Dramaga, Bogor, 16680 Indonesia

**Keywords:** Peranakan Ongole, Nellore, *Bos indicus*, *Bos javanicus*, birth weight

## Abstract

**Background:**

Peranakan Ongole (PO) is a major Indonesian *Bos indicus* breed that derives from animals imported from India in the late 19^th^ century. Early imports were followed by hybridization with the *Bos javanicus* subspecies of cattle. Here, we used genomic data to partition the ancestry components of PO cattle and map loci implicated in birth weight.

**Results:**

We found that *B. javanicus* contributes about 6-7 % to the average breed composition of PO cattle. Only two nearly fixed *B. javanicus* haplotypes were identified, suggesting that most of the *B. javanicus* variants are segregating under drift or by the action of balancing selection. The zebu component of the PO genome was estimated to derive from at least two distinct ancestral pools. Additionally, well-known loci underlying body size in other beef cattle breeds, such as the *PLAG1* region on chromosome 14, were found to also affect birth weight in PO cattle.

**Conclusions:**

This study is the first attempt to characterize PO at the genome level, and contributes evidence of successful, stabilized *B. indicus* x *B. javanicus* hybridization. Additionally, previously described loci implicated in body size in worldwide beef cattle breeds also affect birth weight in PO cattle.

**Electronic supplementary material:**

The online version of this article (doi:10.1186/s12863-015-0229-5) contains supplementary material, which is available to authorized users.

## Background

The humped Ongole or Nellore cattle are indigenous to the Nellore-Ongole region in Prakasam District (Andhra Pradesh State), Southeastern coast of India. Molecular evidence supports a pure *Bos indicus* origin to the modern Indian Ongole population [1]. The history of Ongole cattle is only partially documented, but it is believed to date back to the late Bronze Ages (some 4000 years ago) when pastoral nomad Aryan tribes migrated to India, bringing different types of cattle that rapidly spread throughout the country [2]. These imports probably contributed to the formation of white/gray cattle breeds over the centuries, such as the Ongole. Heat tolerance, disease resilience, and draft power have made Ongole cattle attractive for beef production in low-input systems, which stimulated imports of Ongole bulls to several tropical countries in the late 19^th^ century, including South America [3] and Indonesia.

Ongole cattle importation to Indonesia from the Nellore province of India was carried out by Dutch colonials, with massive imports from 1905 to 1920. In 1912, the Dutch government designated the Sumba Island as the official location for maintaining imported Ongole animals, which led to the formation of the Sumba Ongole (SO) population. From 1915 to 1929, the “Ongolization program” was responsible for distributing SO animals to several regions of Indonesia, including the Java Island, where SO cattle were crossed with local *Bos javanicus* cattle (also known as Java or Banteng cattle), and formed the Ongole-grade or Peranakan Ongole (PO) breed [4, 5]. This historical hybridization is supported by microsatellite and mitochondrial DNA data [6]. To date, PO cattle are one of the most popular breeds in Indonesia, spreading almost evenly throughout the country, ranging from Sumatra, Java and Sulawesi Islands. Yet, the breed remains genetically uncharacterized and poorly selected for production traits.

The uncontrolled use of bulls together with non-systematic breeding has undermined the genetic progress of PO cattle in Indonesia over the years. Consequently breeders have recently started to produce crossbreds between PO and exotic taurine breeds, such as Limousin and Simmental, in an attempt to rapidly improve beef production, thus threatening the conservation of the breed. The identification of quantitative trait loci (QTLs) underlying traits of interest, such as body size and weight, may be of help to encourage breeders to further explore the genetic potential of the breed.

Here, we used a panel of 54,609 SNPs (50k) to genetically characterize PO cattle by comparison with *B. taurus*, *B. indicus*, *B. javanicus* and composite *B. taurus* x *B. indicus* breeds. We aimed at: i) assessing the genetic relationships between PO and other cattle breeds; ii) estimating the contribution of *B. javanicus* to PO breed composition; iii) partitioning the ancestral origins of the *B. indicus* component of the PO genome; iv) map putative *B. javanicus* haplotypes segregating in PO; and 5) investigating whether major QTLs for body size previously found in other breeds also segregate in PO.

## Methods

### Ethical statement

All animal procedures related to PO and BALI samples were approved by the Indonesian Agency for Agricultural Research and Development, Ministry of Agriculture, KKP3N activity 70/PL.220/I.1/3/2014. Genotypes for the remaining breeds were obtained from previously published data [7].

### Genotypes, phenotypes and quality control

A total of 48 Indonesian PO male steers were genotyped for 54,609 SNPs using the Illumina® BovineSNP50 v2 Genotyping BeadChip assay (50K), according to the manufacturer's protocol. These animals were selected based on their extreme phenotypic values for birth weight (used here as a proxy for body size), representing the lower (n = 24, mean = 21.31 ± 1.61 kg) and upper (n = 24, mean = 28.75 ± 2.82 kg) tails of the phenotypic distribution. Additionally, 18 *B. javanicus* animals from the Bali Island (BALI) were genotyped with the 50k panel.

The following Illumina® BovineHD genotypes (HD - 786,799 SNPs) were available from previously published data [7]: European *B. taurus* Holstein (HOL, n = 59), African *B. taurus* N'Dama (NDA, n = 24), Brazilian *B. indicus* of Indian origin Nellore (NEL, n = 35) and Gyr or Gir (GIR, n = 30), admixed *B. indicus* Brahman (BRM, n = 25) and modern *B. taurus* x *B. indicus* composites Santa Gertrudis (SGT, n = 24) and Beef Master (BMA, n = 24). The later two were included because both have *B. taurus* and *B. indicus* contributions to their genomes: Ongole was one of the breeds directly used in the formation of SGT, whereas the creation of the BMA composite was intermediated by the use of Brahman bulls. On the other hand, Brahman is a *B. indicus* breed developed using Gir, Nellore and Guzerat, which has been crossed with *B. taurus* breeds.

PO cattle genotypes were merged with the remaining samples into a single dataset by overlapping the common set of markers between the HD and the 50K panels. Both data sets had genomic coordinates annotated in the UMD v3.1 reference assembly. The final data comprised 287 samples and 49,915 SNPs. Markers and individuals were removed from the dataset using *PLINK v1.07* [8] if they did not present call rate of at least 90 %. We decided to not trim the data by minor allele frequency (MAF) in the breed level, as markers with private alleles may be highly informative in comparative analyses. Instead, we excluded SNPs that were monomorphic across all breeds. Summary statistics such as pairwise F_ST_ and heterozygosity were also computed and made available as supplementary data (Additional file 1).

### Genotype clustering and admixture analysis

Genetic relationships between PO and other worldwide cattle breeds were determined using both distance-based and model-based genotype clustering analyses. In the distance-based method, we performed a Classical Multidimensional Scaling (CMDS) analysis using *PLINK v1.07* and customized scripts in *R v3.1.2* (available at: http://www.r-project.org/). First, a similarity matrix was constructed from the proportion of alleles shared identically by state between each possible pair of samples. Then, CMDS was applied to the similarity matrix, and the first two principal coordinates were used to obtain a two-dimensional graphical representation of relationships among individuals.

*ADMIXTURE v1.23* was used for a model-based unsupervised clustering of individuals via maximum likelihood [9]. We assumed different models where the individuals’ genome could be partitioned in *K* clusters, assuming they may derive from *K* different ancestral populations. We started with a model assuming that genome fragments derive from one of four ancestral populations (*K* = 4): European *B. taurus*, African *B. taurus*, *B. indicus* or *B. javanicus*. Then, we fitted models with increasing number of clusters up to the number of breeds (*K* = 5 to *K* = 9).

Although the model implemented in *ADMIXTURE v1.23* allows for estimating ancestry proportions, it does not provide means to formally test for the presence of admixture. Therefore, we used the *threepop* program of the *TreeMix v1.12* package [10] to compute *f*_3_ statistics [11]. Standard deviation scores (Z-scores) for *f*_3_ were computed for all possible population triplets. Significant admixture was declared when Z < −3. Additionally, *TreeMix v1.12* was also used to generate a maximum likelihood phylogeny with migration edges. The tree was rooted on *B. javanicus* and computed using blocks of 1000 markers. The assumed number of migration events was chosen to match the number of admixed breeds in the model-based clustering analysis.

### Detection of PO haplotypes inherited from *Bos javanicus*

Probability of *B. javanicus* haplotypic ancestry was estimated following Bolormaa et al. (2011) [12]. We estimated haplotype phase and imputed missing genotypes using SHAPEIT2 [13]. Haplotype frequencies were computed for overlapping windows of five consecutive markers using customized scripts in *R*. Then, for each haplotype found in PO, the probability of *B. javanicus* ancestry was estimated as:

$$ \mathit{\mathsf{Pr}}\left(\mathit{\mathsf{B}}\mathit{\mathsf{j}}\right)=\frac{{\mathit{\mathsf{p}}}_{\mathit{\mathsf{B}}\mathit{\mathsf{j}}}}{{\mathit{\mathsf{p}}}_{\mathit{\mathsf{B}}\mathit{\mathsf{j}}}+{\mathit{\mathsf{p}}}_{\mathit{\mathsf{B}\mathsf{i}}}} $$

where ${\mathit{\mathsf{p}}}_{\mathit{\mathsf{B}}\mathit{\mathsf{j}}}$ and ${\mathit{\mathsf{p}}}_{\mathit{\mathsf{Bi}}}$ are the haplotype frequencies in *B. javanicus* and *B. indicus*, respectively. Haplotype frequency in *B. javanicus* was estimated using BALI samples. For *B. indicus*, we used NEL and GIR samples to compute haplotype frequencies. Haplotypes with a frequency of at least 10 % in PO cattle presenting ${\mathit{\mathsf{p}}}_{\mathit{\mathsf{B}}\mathit{\mathsf{j}}} > \mathsf{0.8}$ in all comparisons were considered as candidate identical-by-descent (IBD) *B. javanicus* haplotypes.

Haplotypes were further filtered and divided into three groups: group A, comprising haplotypes that were absent in *B. indicus* breeds but present in *B. javanicus*; group B, including haplotypes with high frequency in PO and *B. javanicus* (>10 %) but low frequency in *B. indicus* (<5 %); group C, haplotypes with moderate-to-high frequencies in *B. indicus* (>5 %) but that were more frequent in *B. javanicus;* and group D, including all remaining haplotypes. Group A represented a proxy for *B. javanicus* private haplotypes. Group B was likely to contain haplotypes derived from *B. javanicus* that are identical-by-state (IBS) with *B. indicus* rare haplotypes. Group C was similar to Group B, except that the IBS haplotypes in *B. indicus* had higher frequencies. Additionally, nearly fixed (>80 %) *B. javanicus* haplotypes were considered candidates under selection, regardless of the groups mentioned above.

### Genome-wide mapping of loci affecting PO birth weight

Putative QTLs for birth weight were detected using the following regression model:

$$ \mathit{\mathsf{y}}={1}_n\mathit{\mathsf{\mu}}+\mathit{\mathsf{x}}\mathit{\mathsf{b}}+\mathit{\mathsf{g}}+e $$

where $\mathit{\mathsf{y}}$ is the vector of birth weights, 1_*n*_ is a vector of 1's, $\mathit{\mathsf{\mu}}$ is the overall mean, $\mathit{\mathsf{x}}$ is an incidence vector for birth season, $\mathit{\mathsf{b}}$ is the fixed effect of birth season, $\mathit{\mathsf{g}}=\mathit{\mathsf{Z}}\mathit{\mathsf{a}}$ is the vector of random direct genomic values (i.e., sum of the effects of genome-wide markers), $\mathit{\mathsf{Z}}$ is the matrix of centered genotypes, $\mathit{\mathsf{a}}$ is the vector of random marker effects, and *e* is the vector of residual effects.

de Los Campos et al. (2009) [14] demonstrated that this model can be re-parameterized by $\mathit{\mathsf{g}}=\mathit{\mathsf{K}}\mathit{\mathsf{c}}$, where $\mathit{\mathsf{K}}$ is the kernel matrix of additive genetic relationships between pairs of individuals, $\mathit{\mathsf{c}}$ is a random vector distributed as $\mathit{\mathsf{N}}\left(0,{\mathit{\mathsf{K}}}^{-1}{\mathit{\mathsf{\sigma}}}_{\mathit{\mathsf{c}}}^2\right)$, and ${\mathit{\mathsf{\sigma}}}_{\mathit{\mathsf{c}}}^2$ is the variance attributed to $\mathit{\mathsf{c}}$. The additive kernel matrix can be computed as $\mathit{\mathsf{K}}=\mathit{\mathsf{Z}}\mathit{\mathsf{D}}\mathit{\mathsf{Z}}\hbox{'}\mathit{\mathsf{q}}$ [15], where the scaling parameter $\mathit{\mathsf{q}}$ is $1/{\displaystyle \sum {2\mathrm{p}}_{\mathit{\mathsf{i}}}}\left(1-{\mathit{\mathsf{p}}}_{\mathit{\mathsf{i}}}\right),\ {\mathit{\mathsf{p}}}_{\mathit{\mathsf{i}}}$ is the reference allele frequency at marker $\mathit{\mathsf{i}}$, and $\mathit{\mathsf{D}}$ is a diagonal matrix of marker weights. This parametrization avoids fitting a model with as many predictors as markers by fitting as many predictors as samples. Marker effects can be back-transformed from estimates $\widehat{\mathit{\mathsf{c}}}$ as in the Genomic Best Linear Unbiased Predictor (GBLUP) method [16]:

$$ \widehat{\mathit{\mathsf{a}}}=\mathit{\mathsf{q}}\mathit{\mathsf{D}}\mathit{\mathsf{Z}}\hbox{'}{\left(\mathit{\mathsf{Z}}\mathit{\mathsf{D}}\mathit{\mathsf{Z}}\hbox{'}\mathit{\mathsf{q}}\right)}^{-1}\widehat{\mathit{\mathsf{g}}} $$

Under the assumption of equal variance across markers, $\mathit{\mathsf{D}}=\mathit{\mathsf{I}}$ and

$$ \widehat{\mathit{\mathsf{a}}}=\mathit{\mathsf{q}}\mathit{\mathsf{Z}}\hbox{'}{\mathit{\mathsf{K}}}^{-1}\widehat{\mathit{\mathsf{g}}} $$

From the definitions above, $\widehat{\mathit{\mathsf{g}}}=\mathit{\mathsf{K}}\widehat{\mathit{\mathsf{c}}}$, then:

$$ \widehat{\mathit{\mathsf{a}}}=\mathit{\mathsf{q}}\mathit{\mathsf{Z}}\hbox{'}{\mathit{\mathsf{K}}}^{-1}\mathit{\mathsf{K}}\widehat{\mathit{\mathsf{c}}} $$

Since ${\mathit{\mathsf{K}}}^{-1}\mathit{\mathsf{K}}=\mathit{\mathsf{I}}$, we have:

$$ \widehat{\mathit{\mathsf{a}}}=\mathit{\mathsf{q}}\mathit{\mathsf{Z}}\hbox{'}\widehat{\mathit{\mathsf{c}}} $$

Model parameters were estimated using the Gibbs sampling algorithm implemented in the *BGLR v1.0.3* package in *R v.3.1.2* [17]. Normal priors were assigned to random effects and flat priors were assigned to the overall mean and birth season. Variance components were assumed a priori scaled inverse chi-squared distributed. A single Markov chain with a length of 1,000,000 iterations was used. The burn-in period was set at 10,000 iterations and the thinning interval at 100 iterations. Only polymorphic autosomal SNPs presenting Fisher's exact test p-value for Hardy-Weinberg equilibrium (HWE) greater than 1 × 10^−20^ were included in this analysis. Posterior samples for the variance explained by genome-wide SNPs were obtained as $\frac{{\widehat{\sigma_c}}^2}{{\widehat{\sigma_c}}^2+{\widehat{\sigma_e}}^2}$ and the point estimate was derived from the average of these samples. The 95 % credible interval was defined as the 2.5 % and 97.5 % percentiles of the posterior distribution.

The variance attributed to overlapping chromosome segments encompassed by 20 consecutive SNPs was computed as $\mathit{\mathsf{v}}\mathit{\mathsf{a}}\mathit{\mathsf{r}}\left({\displaystyle \sum_{\mathit{\mathsf{i}}=1}^{20}{\mathit{\mathsf{z}}}_{\mathit{\mathsf{i}}}}{\widehat{\mathit{\mathsf{a}}}}_{\mathit{\mathsf{i}}}\right)$ [18]. As this analysis was underpowered due to small sample size (n = 48), we considered candidate QTLs only the top scoring SNP windows with variance above $10\mathit{\mathsf{I}}\mathit{\mathsf{Q}}\mathit{\mathsf{R}}+\mathit{\mathsf{Q}}\mathsf{3}$ [19], where $\mathit{\mathsf{I}}\mathit{\mathsf{Q}}\mathit{\mathsf{R}}$ and $\mathit{\mathsf{Q}}\mathsf{3}$ are the interquartile range and the third quartile of the distribution of SNP window variances, respectively.

## Results and discussion

### Genetic relationships between Peranakan Ongole and other cattle breeds

**Fig. 1:**
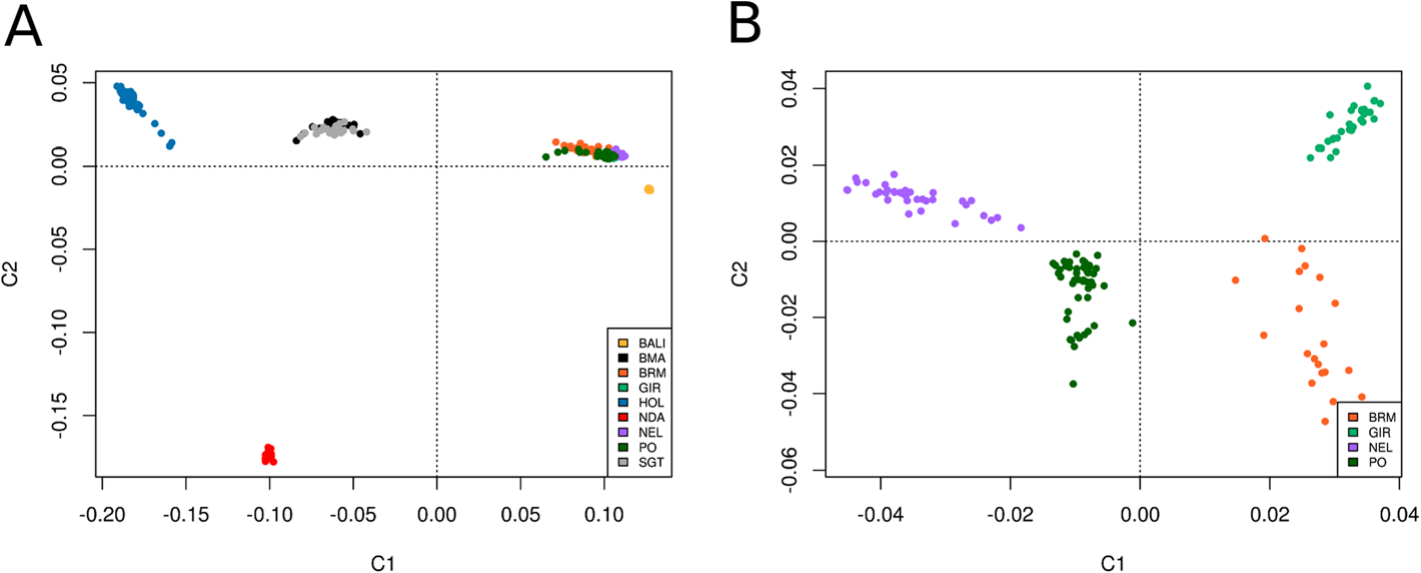
Multidimensional scaling analysis. When all breeds are simultaneously analyzed (**a**), the differences between European *B. taurus* (HOL), African *B. taurus* (NDA) and *B. indicus* breeds (PO, BRM, GIR and NEL) are well demonstrated. However, *B. indicus* breeds are poorly distinguishable due to ascertainment bias. The analysis of *B. indicus* breeds alone (**b**) resolves the relationships among BRM, GIR, NEL and PO cattle, highlighting a closer proximity between the later two. See Material and Methods for breed abbreviations

When all breeds were analyzed simultaneously using CMDS (Fig. 1a), the first coordinate (C1, x-axis) explained the genetic differences between *B. taurus, B. indicus* and *B. javanicus*. The second coordinate (C2, y-axis) separated breeds by geographical origin, explaining genetic differences between African and non-African cattle. These findings are consistent with the previously reported clustering behavior of 50k genotypes in worldwide cattle breeds [20, 21]. As *B. indicus* breeds were poorly separated in comparison to the *B. taurus* breeds as a result of ascertainment bias [22] (see Additional file 1), the relationships among *B. indicus* breeds were assessed by re-running the analysis without *B. taurus* and *B. javanicus* genotypes (Fig. 1b). This analysis revealed that GIR, BRM, PO and NEL cluster as distinct populations, with PO and NEL exhibiting greater similarity. This is not unexpected, provided PO and NEL were believed to derive from the same ancestral population.

### Ancestry components in the Peranakan Ongole genome

**Fig. 2:**
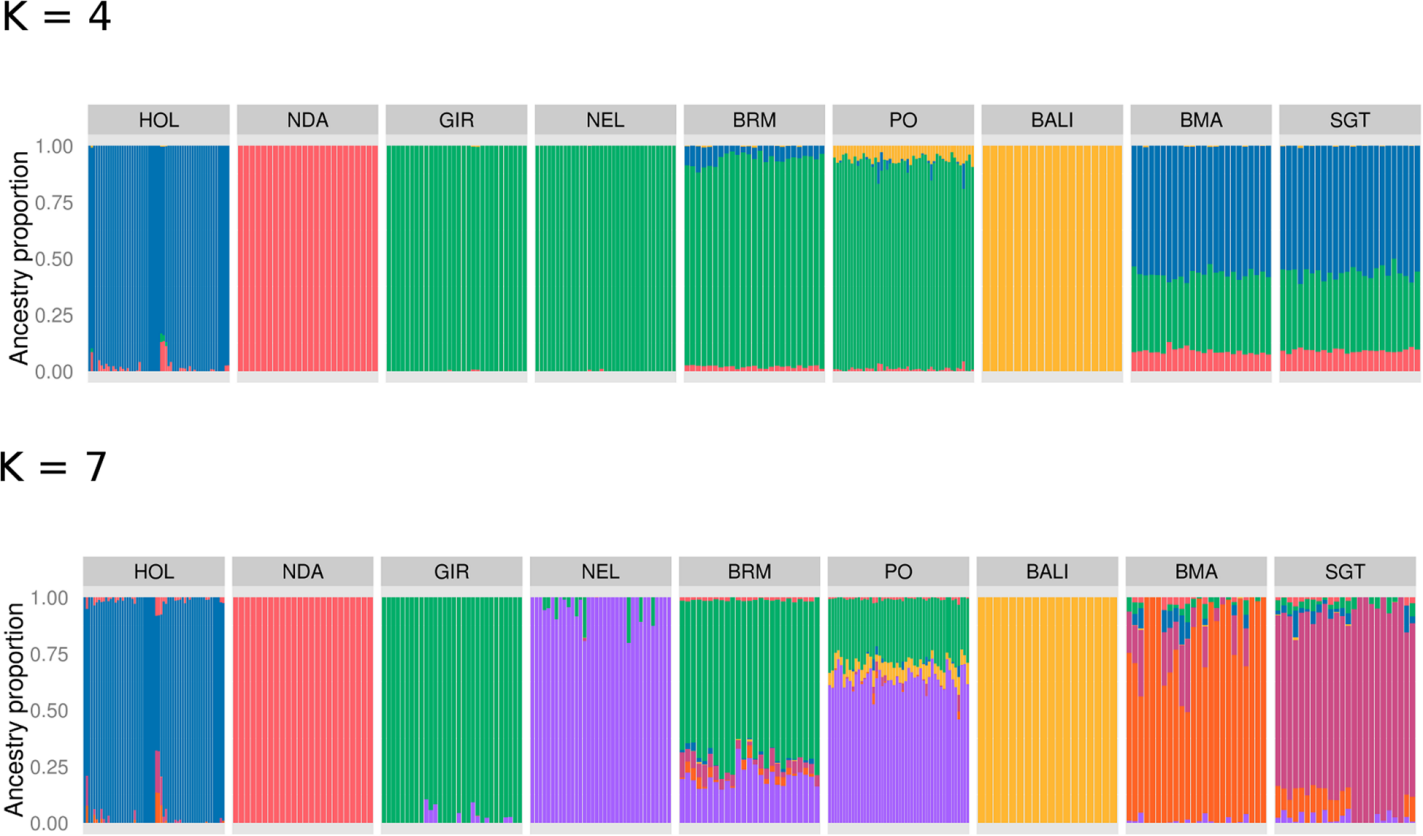
Model-based clustering of cattle breeds assuming different numbers of ancestral populations (*K*). Each individual is represented by a vertical bar that can be partitioned into colored fragments with length proportional to cluster contribution. *K* = 4 approximates the ancestral European *B. taurus*, African *B. taurus*, *B. indicus* and *B. javanicus* populations. *K* = 7 distinguishes between the two zebu ancestors that generated GIR and NEL, and PO exhibits contributions from both ancestral populations. See Material and Methods for breed abbreviations

Results from the model-based clustering analysis are found in Fig. 2. When *K* = 4 was assumed, European *B. taurus*, African *B. taurus*, *B. javanicus* and *B. indicus* breeds were assigned to different clusters. SGT and BMA presented an average *B. indicus* contribution of 34 %, whereas BRM exhibited an average of 6 % of *B. taurus* ancestry. Interestingly, backcrossing to Ongole bulls over the course of 100 years was not capable of eliminating all *B. javanicus* introgression in PO cattle, which presented a mean *B. javanicus* ancestry of approximately 6 %. Historical hybridization with *B. javanicus* cattle seems to also extend to other Indonesian *B. indicus* populations, such as the Brebes [21] and Madura [21, 23] breeds. This is in contrast with the historical *B. taurus* introgression in NEL and GIR [3], which seems to have been consistently eliminated by intensive backcrossing [20, 21, 24]. This suggests that either *B. javanicus* haplotypes were kept by selective forces, or backcrossing in Indonesia was not as strong as in Brazil, and *B. javanicus* haplotypes are just drifting in PO cattle.

*Bos indicus* and *B. taurus* have karyotypes consisting of 29 pairs of acrocentric autosomes, whereas *B. javanicus* has 25 pairs of acrocentric and two pairs of submetacentric autosomes. Based on cross-species fluorescence *in-situ* hybridization analysis, Ropiquet and colleagues [25] suggested that the bi-armed autosomes in *B. javanicus* are equivalent to Robertsonian translocations of autosomes 1–29 and 2–28 in *B. taurus*. It is still unclear how the first generations of Indonesian *B. indicus* × *B. javanicus* hybrids coped with chromosome number imbalance, but the preservation of sequence homology between these species of cattle seems to have guaranteed successful hybridization.

The *f*_3_ statistics [11] provided further support for the evidence of *B. javanicus* hybridization in the PO genome. As expected, only SGT, BMA, BRM and PO presented Z-scores lower than −3, suggesting no significant mixture in HOL, NDA, NEL, GIR and BALI. The lowest scores for SGT (Z = −10.12) and BMA (Z = −9.51) were obtained with the comparisons HOL-NEL and HOL-GIR, respectively, reproducing the known crossbreeding in these populations. In the case of BRM, the lowest score (−9.18) was obtained by contrasting with HOL-GIR, also highlighting the *B. taurus* introgression in this breed. The mixture between *B. javanicus* and *B. indicus* in PO was well supported with a score of −17.88 for the sister group BALI-NEL.

We carried out further admixture analyses assuming different numbers of ancestral populations in order to dissect the *B. indicus* component of the PO cattle genome. In spite of over 100 years of isolation, NEL and PO share a very similar history of importation [3] and are deemed to derive from the same Indian Ongole population. Therefore, it was expected that all *B. indicus* ancestry in PO pertained to the same origin of NEL. Surprisingly, at *K* = 7 (Fig. 2), GIR separated from NEL, revealing that PO has contributions from both ancestral pools, with an average of 20.3 % of the PO genome descending from the same ancestral population that originated GIR. Two hypotheses can be formulated from this finding: 1) the first imports of Ongole to Brazil comprised purebred animals, whereas Indonesian imports comprised admixed animals; 2) both Brazilian and Indonesian imports included admixed animals, and systematic breeding in Brazil promoted selection against the GIR component.

**Fig. 3:**
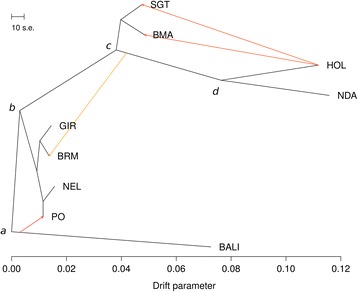
Estimated phylogenetic tree of cattle breeds. The scale bar represents 10 times the average standard error of the estimated entries in the sample covariance matrix. Migration edges were heat-colored according to the weight of contribution from the parental migrant population. Migration edges show *B. javanicus* hybridization into PO cattle, and European *B. taurus* introgression into SGT, BMA and BRM. Vertex *a* approximates the divergence between *Bos primigenius* and *B. javanicus*. Vertex *b* bifurcates the ancestral *B. primigenius* into the ancestors of *B. taurus* and *B. indicus*. Vertex *c* is an artificial bifurcation of modern composite breeds SGT and BMA. Vertex *d* approximates the divergence between European and African *B. taurus* cattle

Together, these results predicted that the phylogenetic analysis should cluster PO to the *B. indicus* clade, and a migration edge should be drawn from the *B. javanicus* branch towards PO. Therefore, we constructed a maximum likelihood tree using *TreeMix* [10], assuming four migration events representing mixtures in SGT, BMA, BRM and PO. Indeed, the estimated phylogeny behaved as predicted (Fig. 3). As expected, the remaining three estimated migrations were European *B. taurus* introgressions into SGT, BMA and BRM.

### *Bos javanicus* haplotypes in Peranakan Ongole

We examined a total of 113,665 PO haplotypes with a frequency of at least 10 %. From these, 8010 haplotypes presented $\mathit{\mathsf{Pr}}\left(\mathit{\mathsf{B}}\mathit{\mathsf{j}}\right) > \mathsf{0.8}$ (Additional file 2). These stringent frequency and probability thresholds were adopted in order to minimize the false positive rate in our sample, considering that low frequency haplotypes could have been generated due to genotyping or phasing errors, and because we were particularly interested in finding high frequency private *B. javanicus* haplotypes in PO cattle. We considered only NEL and GIR as reference *B. indicus* because these breeds are representative of the ancestral populations that originated PO, and because BRM presented *B. taurus* introgression in the clustering analysis. Remarkably, the proportion of putative *B. javanicus* haplotypes in respect to all detected haplotypes was approximately 7 %, which is consistent with the average ancestry estimated from the admixture analysis.

A total of 779, 1480, 3286 and 2465 haplotypes were categorized in groups A (private *B. javanicus* haplotypes), B (*B. javanicus* haplotypes IBS with *B. indicus* rare haplotypes), C (*B. javanicus* haplotypes IBS with *B. indicus* common haplotypes), and D (remaining candidates), respectively (Additional file 1). Although groups A and B were of primary interest, these categories can also emerge from the ascertainment bias of the SNP panel. Group C is less prone to false positives induced by SNP bias, but is also more susceptible to ancestry mis-assignment because frequent IBS haplotypes are found in *B. javanicus* and *B. indicus*.

Only two genomic regions presented nearly fixed *B. javanicus* haplotypes (frequency > 80 %): 5:106.1-106.5Mb and 16:33.0-33.4Mb. The highest five markers haplotype frequency within the chromosome 5 region (97.9 %) was also fixed in *B. javanicus,* but absent in *B. indicus*. This segment contained four protein-coding genes: fibroblast growth factor 6 (*FGF6*), fibroblast growth factor 23 (*FGF23*), fructose-2,6-bisphosphatase (*FR2BP*, also known as TP53-induced glycolysis and apoptosis - *TIGAR*), and cyclin D2 (*CCND2*). The chromosome 16 region contained nearly fixed haplotypes in PO and *B. javanicus* (>91 %), but with modest frequencies in *B. indicus* (<12 %). Five protein-coding genes mapped to this region: EF-hand calcium binding domain 2 (*EFCAB2*), heterogeneous nuclear ribonucleoprotein U (*HNRNPU*), COX20 *(FAM*36A), peptidase domain containing 1 (*PPPDE1*) and human chromosome 1 open reading frame 101 (*C1ORF101*). Additionally, one small nucleolar RNA and one U6 spliceosomal RNA also mapped to this chromosome 16 domain.

Besides the possibility of false positives due to ascertainment bias, these haplotypes may have achieved nearly fixation either because an advantageous *B. javanicus* allele in this chromosome segment has been subjected to positive selection in PO, or because this *B. javanicus* haplotype hitchhiked with a selected *B. indicus* variant. The small number of nearly fixed *B. javanicus* haplotypes suggests that most of these are just drifting in PO, but we also do not discard balancing selection.

### Candidate QTLs for birth weight in Peranakan Ongole

The genotype filters reduced the SNP panel to 34,482 markers, which were estimated to explain 33.5 % of the variance in birth weights, with a 95 % credible interval of 12.0 % - 67.6 %. These estimates must be taken with caution, as reliable variance components inference often requires the analysis of thousands of records. A clear limitation of the QTL mapping analysis presented here is the use of a small sample size (n = 48), rendering our investigation prone to high false discovery rates and limited to the detection of major QTLs. Nevertheless, we extensively compared the results obtained here with the literature in order to determine whether some of the detected loci have been previously associated with body size traits in other cattle breeds.

**Fig. 4:**
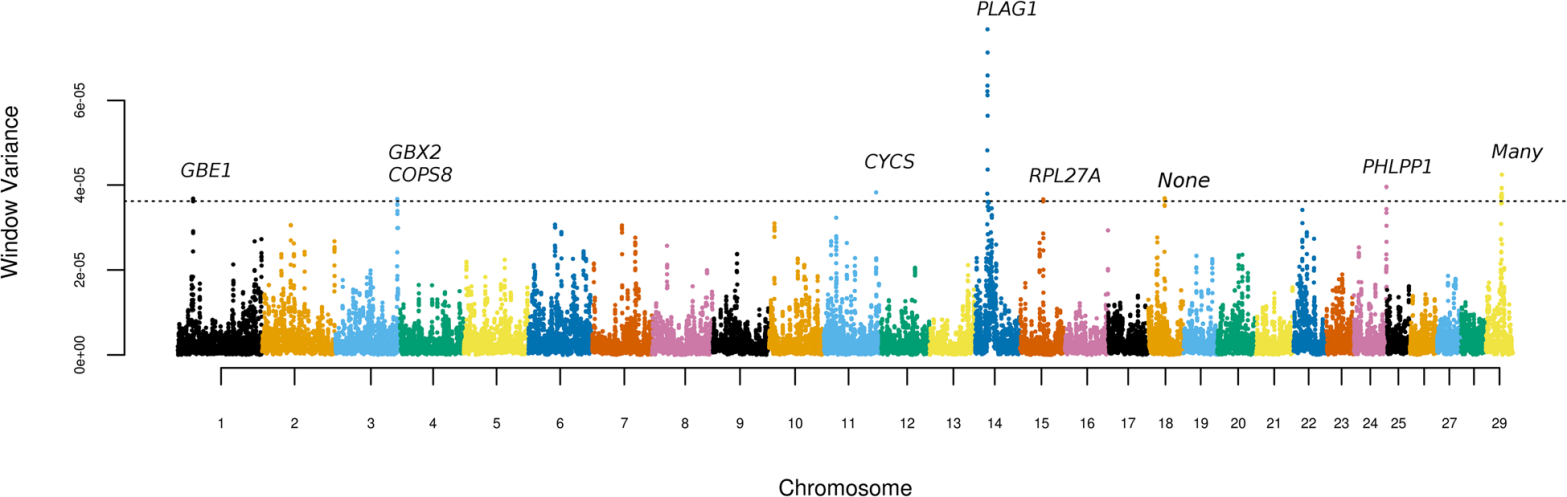
Manhattan plot of birth weight variance attributed to overlapping windows of 20 consecutive SNPs. The dashed horizontal line represents the $10\mathit{\mathsf{I}}\mathit{\mathsf{Q}}\mathit{\mathsf{R}}+\mathit{\mathsf{Q}}\mathsf{3}$ threshold, where $\mathit{\mathsf{I}}\mathit{\mathsf{Q}}\mathit{\mathsf{R}}$ and $\mathit{\mathsf{Q}}\mathsf{3}$ are the interquartile range and the third quartile of the distribution of SNP window variances, respectively

A total of 34,221 windows of 20 consecutive SNPs were inspected, and in spite of the small number of PO samples, positional candidates for birth weight were found on chromosomes (CHR) 1, 3, 11, 14, 15, 18, 24 and 29 (Fig. 4). Remarkably, the majority of these candidate loci have already been described as associated with body size in beef cattle.

The positional candidate locus on CHR14 (peaking at 25 Mb) maps to the *PLAG1* (pleiotropic adenoma gene 1) chromosomal domain, and was previously found to be associated with birth weight in NEL cattle [26], as well as with a number of body size [27–30] and reproductive traits [19, 31, 32] in several *B. taurus* and *B. indicus* breeds. The mechanism by which *PLAG1* may affect fetal growth and reproduction is most likely associated with the trans-acting regulation of the expression of insulin-like growth factors, particularly *IGF2* [33].

In Brahmans, the C allele of rs109231213 in the vicinity of *PLAG1* is associated with positive effects on body size but negative effects on fertility, and also presents evidence of selection and *B. taurus* origin [29]. We hypothesized that a similar phenomenon could underly birth weight variance in PO, namely selective pressures on the *PLAG1* region potentially involving *B. javanicus* haplotypes.Interestingly, this CHR14 segment indeed presented large LD blocks (data not shown). High LD in this chromosome domain has also been observed in NEL [19], and a possible event of balancing selection has been hypothesized in that breed [26]. However, this long range LD can be partially explained by our sampling strategy, which privileged extremes from the phenotypic distribution. Furthermore, we found no evidence of differential effects between *B. javanicus* and *B. indicus* haplotypes for this or any other candidate QTLs (data not shown), suggesting that variants of *B. indicus* origin are responsible for differences in birth weight in PO.

The putative QTL on CHR3 (116.4 to 116.8 Mb) is in the vicinity of associations for ribeye area in Shorthorn [28] and calf size in Holstein cattle [34], and contains genes that are involved in early embryo development and growth, such as gastrulation brain homeobox 2 (*GBX2*) and constitutive photomorphogenic homolog subunit 8 (*COPS8*). The CHR15:44.6-45.0 Mb region is nearby the ribosomal protein L27a gene (*RPL27A*) and coincides with QTLs for mature weight and ribeye area in Hereford [28] and mature height, ribeye area and carcass weight in Angus [35]. The 30.9 to 32.2 Mb region on CHR18 is in a gene desert but flanks QTLs for longissimus dorsi area and body weight in Angus [35], and carcass weight in Angus x Brahman crosses [36]. In contrast, the locus on CHR29 (29.2 to 30.7 Mb) contains 24 protein-coding genes, and also overlaps QTLs for carcass, weaning and yearling weights in Maine-Anjou cattle [28]. Interestingly, the adoption of the highly stringent Bonferroni correction prevented the detection of this QTL in a genome-wide association study for birth weight in NEL [26].

The candidate QTLs on CHR1 (28.9 to 29.2 Mb), CHR11 (97.8 to 98.5 Mb), and CHR24 (61.4 to 62.0 Mb) did not overlap any previously identified QTLs for body size or related traits, and are either novel QTLs or false discoveries. However, these loci harbor interesting candidate genes. For instance, the CHR1 region shelters the 1,4-alpha-glucan branching enzyme gene (*GBE1*), responsible for increasing the solubility of glycogen molecules to allow its accumulation in the liver and muscle cells, and could be involved in somatic growth and muscle development. The CHR11 region contains the somatic cytochrome c gene (*CYCS*), previously shown to affect embryonic growth [37]. Finally, the CHR24 locus shelters the PH domain and leucine rich repeat protein phosphatase 1 gene (*PHLPP1*). Disruption of this gene in mice has been recently found to lead to decreased bone mass [38].

## Conclusions

We found molecular evidence of *B. javanicus* × *B. indicus* hybridization in the genomes of Peranakan Ongole steers, consistent with historical records. Haplotype analyses detected only two candidate *B. javanicus* haplotypes that may have been subjected to positive selection, suggesting that the majority of the *B. javanicus* contribution is either drifting or under balancing selection. We also estimated that the *B. indicus* ancestry component of Peranakan Ongole animals derived from two distinguishable genetic pools that are closely related to Brazilian Nellore and Gir cattle. Quantitative trait loci underlying body size in other beef cattle populations were shown to also segregate in Peranakan Ongole cattle, representing an opportunity for selection and improvement of this breed.

### Availability of supporting data

The original data sets supporting the results of this article are available in Additional file 3. They are accessible by password request from the authors.

## Additional files

Additional file 1:
**Heterozygosity and F**_**ST**_ **analysis.**
Additional file 2:
**List of putative** ***Bos javanicus*** **haplotypes in Indonesian Peranakan Ongole.**
Additional file 3:
**Supporting data (temporary browsable access):** **https://drive.google.com/file/d/0B4MpcTN6ypdyOXhGaE9CXzFvTHc/view?usp=sharing** **.**

## Abbreviations

PO: Peranakan Ongole
SO: Sumba Ongole
NEL: Nellore
GIR: Gyr or Gir
HOL: Holstein
NDA: N'Dama
SGT: Santa Gertrudis
BMA: Beefmaster
CMDS: Classical multidimensional scaling
SNP: Single-nucleotide polymorphism
CHR: Chromosome
MAF: Minor allele frequency
50K: Illumina® BovineSNP50 v2 Genotyping BeadChip assay
HD: Illumina® BovineHD BeadChip assay
QTL: Quantitative trait locus
HWE: Hardy-Weinberg equilibrium
